# Outcomes in patients requiring intensive care unit (ICU) admission after emergency laparotomy: A retrospective study

**DOI:** 10.1111/aas.14103

**Published:** 2022-06-19

**Authors:** Aura T. Ylimartimo, Marjo Koskela, Sanna Lahtinen, Timo Kaakinen, Merja Vakkala, Janne Liisanantti

**Affiliations:** ^1^ Medical Research Center of Oulu Research Group of Surgery, Anesthesiology and Intensive Care Medicine Oulu Finland; ^2^ Department of Surgery Oulu University Hospital Oulu Finland; ^3^ Department of Anesthesiology Oulu University Hospital Oulu Finland

**Keywords:** emergency laparotomy, emergency surgery, intensive care unit (ICU), mortality

## Abstract

**Purpose:**

Outcomes after emergency laparotomy (EL) are poor. These patients are often admitted to an intensive care unit (ICU). This study explored outcomes in patients who were admitted to an ICU within 48 h after EL.

**Materials and Methods:**

This retrospective single‐center registry study included all patients over 16 years of age that underwent an EL and were admitted to an ICU within 48 h after surgery in Oulu University Hospital, Finland between January 2005 and May 2015. Survival was followed until the end of 2019.

**Results:**

We included 525 patients. Hospital mortality was 13.3%, 30‐day mortality was 17.3%, 90‐day mortality was 24.2%, 1‐year mortality was 33.0%, and 5‐year mortality was 59.4%. Survivors were younger (57 [45–70] years) than the non‐survivors (73 [62–80] years; *p* < .001). According to the Cox regression model, death during the follow‐up was associated with age, APACHE II‐score, lower postoperative CRP levels and platelet count of the first postoperative day, and the admission from the post‐anesthesia care unit (PACU) to the ICU instead of direct ICU admission.

**Conclusion:**

Age, high APACHE II‐score, low CRP and platelet count, and admission from the PACU to the ICU associated with mortality after EL in patients admitted to an ICU within 48 h after EL.

**Editorial Comment**

Decisions on postoperative ICU admission following emergency laparotomy involve both surgeons and intensivists. Conventional prognosis factors indicate high risk for unfavorable outcomes. This long‐time follow‐up underlines the difficulties involved. A take‐home message is that an optimal care and decision‐making both preoperatively and postoperatively are in the best interest of these patients.

## 1 INTRODUCTION

Emergency laparotomy (EL) is among the most common surgical operations. Typically, patients undergoing EL are elderly with several comorbidities.^1^, ^2^, ^3^, ^4^ Previous studies have shown that emergency surgery is associated with high morbidity and mortality.^1^, ^2^, ^4^, ^5^, ^6^, ^7^ The reported 30‐day mortality rates have varied between 11% and 20%; moreover, up to 30% of the patients have experienced major postoperative complications.^8^ Various tools for identifying the high‐risk patients have been introduced, but none have been optimal.^9^

Due to the high rate of postoperative complications and the high mortality associated with EL, premeditated immediate postoperative intensive care unit (ICU) admissions are common. The previous studies have shown that the postoperative ICU care and standardized perioperative protocols for high‐risk abdominal surgery may reduce mortality and morbidity after an EL.^10^, ^11^, ^12^

In the present study, we explored outcomes in patients admitted to an ICU within 48 h of EL and perioperative factors associated with death during the follow‐up period.

## 2 MATERIALS AND METHODS

This retrospective cohort study was conducted in Oulu University Hospital, in Oulu, Finland. The study was approved by the hospital administration (reference number 66/2018). Data were collected from the electronic medical records, anesthesia charts, and the ICU patient management system's database (Centricity Clinical Care Clinisoft, GE Healthcare). Due to the retrospective study design and according to the local protocol, no statement from the Ethics Committee was required.

All patients (*N* = 525) had undergone an EL and were admitted to an ICU 48 h after surgery between 1 January 2005 and 20 May 2015. The types of operations performed are listed in Table 1.

**TABLE 1:** Demographics of 525 patients admitted to the ICU within 48 h of the EL

Characteristic	Survivors *N* = 213	Non‐survivors *N* = 312	*p*‐value
Age, years	57 (45–70)	73 (62–80)	<.001
Gender, male	112 (52.6)	186 (59.6)	.110
Operation duration (min)	204 (155–263)	195 (155–244)	.179
ASA^a^	4 (3–4)	4 (3–4)	.017
ASA 1	3 (1.5)	1 (0.3)	.149
ASA 2	19 (9.5)	9 (3.0)	.002
ASA 3	65 (32.5)	92 (30.5)	.630
ASA 4	89 (44.5)	160 (53.0)	.063
ASA 5	24 (12.0)	40 (13.2)	.682
Operation diagnosis			
Malignancy/tumor	5 (2.3)	21 (6.7)	.023
Colon/rectum malignancy	3 (1.4)	13 (4.2)	.071
GI malignancy	3 (1.4)	15 (4.8)	.036
GI ulcer	7 (3.3)	25 (8.0)	.026
Hernia	12 (5.6)	19 (6.1)	.830
Diverticulitis/colitis	12 (5.6)	30 (9.6)	.099
Ileus/occlusion	30 (14.1)	44 (14.1)	.995
Peritonitis	20 (9.4)	24 (7.7)	.491
Vascular cause	14 (6.6)	32 (10.3)	.143
HBP	16 (7.5)	10 (3.2)	.026
Other GI diseases	21 (9.9)	34 (10.1)	.703
Injury	23 (10.8)	1 (0.3)	<.001
Other rare causes	1 (0.5)	14 (4.5)	.007
Postoperative complication	52 (24.4)	58 (18.6)	.107
Operation type			
Abdominal wall, mesentery, peritoneum and greater omentum	95 (44.6)	133 (42.6)	.654
Upper GI tract	11 (5.2)	22 (7.1)	.382
Small intestine and colorectal surgery	88 (41.3)	145 (46.5)	.243
HBP	6 (2.8)	0	.003
GI complication	13 (6.1)	12 (3.8)	.233

*Note*: Values are the number (%) or the median (25th–75th percentiles), as indicated.

Abbreviations: ASA, American Society of Anesthesiologists; GI, gastrointestinal; HPB, hepatopancreaticobiliary.

^a^ Missing data n = 13/10.

There is a broad definition of EL, from a laparotomy performed in an unstable patient to include also stable patients. For example, EL for diverticulitis perforation is urgent and patient may be stable or instable. The exclusion criteria were as follows: age under 16 years old, urgent or emergency cholecystectomy or appendectomy, emergency or urgent laparotomies due to gynecological or trauma‐related causes, patients who came for the EL from the ICU and patients admitted to the ICU more than 48 h after an EL.

The setting is a tertiary academic hospital providing 24/7 care for population of 740,000 within the hospital district. According to the local protocol, patients are admitted to the ICU after a high‐risk surgery in case of severe organ dysfunctions or if the expected initial need of postoperative care is longer than 24 h. Otherwise, the patients are admitted to the post‐anesthesia care unit (PACU), from where they move to the surgical ward when the standard local discharge criteria are met. There are 12 PACU beds and 26 ICU beds in the hospital. The need for an ICU admission in surgical patients is assessed before surgery is completed.

The following data were collected: age, sex, diagnosis, type and duration of the operation, time from the end of the operation to the ICU admission (delay), ICU length of stay (LOS), and hospital LOS. The severity of disease was assessed with the Acute Physiology and Chronic Health Evaluation II (APACHE II) and the sepsis‐related organ failure assessment (SOFA). The American Society of Anesthesiologists classification (ASA) was used to estimate the patient's preoperative risk. Postoperative levels of albumin, leukocytes, platelets, hemoglobin, and C‐reactive protein (CRP) were obtained from the ICU patient data management system's database. The date of death was retrieved from the hospital's medical records to assess the in‐hospital, 30‐, 90‐day, 1‐, and 5‐year mortality rates. Patients were followed until the end of 2019 for the long‐term survival analysis.

Due to the retrospective study design, we did not perform a power calculation to assess the sample size. Statistical analyses were performed with IBM SPSS statistics 27 software (IBM SPSS Statistics for Windows, Version 27.0). Categorical data are presented as the number (*n*) and percentage (%). Continuous variables are expressed as the median and 25th and 75th percentiles (25th–75th). Comparisons were performed with Pearson's chi‐square for proportional data and the nonparametric Mann–Whitney test for continuous data. Cox regression analyses were used to estimate the risk factors for death. Two‐tailed *p* < .05 were considered statistically significant.

## 3 RESULTS

This study included 525 patients. Of those, 312 (59.4%) died during the follow‐up. The patient demographics are presented in Table 1.

The survivors were younger than the non‐survivors. Malignancy and GI ulcer as operation diagnoses were more common in non‐survivors.

The non‐survivors had higher Apache II and SOFA scores, and lower first postoperative day platelet count and CRP values. The hospital LOS was longer in the survivors compared to the non‐survivors. The non‐survivors were primarily more often admitted to the post‐anesthesia care unit (PACU) with a later admission to an ICU (27.6% vs 19.6%, *p* = .029) (Table 2).

**TABLE 2:** Outcomes of 525 patients admitted to the ICU within 48 h of the EL

Outcomes	Survivors *N* = 213	Non‐survivors *N* = 312	*p*‐value	Missing
SAPS II	31 (26–39)	40 (32–48)	<.001	2.0
Apache II score	14 (10–18)	18 (14–22)	<.001	2.2
SOFA score on admission	5 (3–7)	6 (3–8)	.008	10.30
SOFA, maximum score	6 (4–9)	8 (5–10)	<.001	0.2
Cumulative TISS score	127 (71–258)	124 (80–238)	.996	0.0
Median TISS score/day	7 (4–13)	8 (4–20)	.040	49.110
CRP, POD1	202 (133–278)	177 (109–265)	.047	4.5
Hemoglobin, POD1	97 (87–105)	98 (90–108)	.178	4.5
Platelet count, POD1	211 (141–294)	190 (119–281)	.032	4.5
Leukocyte, POD1	11.1 (5.7–17.1)	11.3 (6.6–16.6)	.883	4.5
Albumin, POD1	26 (22–32)	27 (22–32)	.957	139.203
Direct admission to ICU	137 (64.3)	186 (59.6)	.277	
Indirect admission to ICU	41 (19.2)	86 (27.6)	.029	
Admission from ward to ICU within 48 h of EL	35 (16.4)	40 (12.8)	.246	
ICU LOS (days)	2.7 (1.4–5.5)	2.4 (1.3–5.2)	.292	
Hospital LOS (days)	20 (11–35)	17 (8–30)	.023	49.110
Invasive ventilation in ICU	164 (77.0)	257 (82.4)	.129	
Duration of invasive ventilation (h)	18.0 (7.4–60.3)	17.0 (5.0–44.2)	.429	

*Note*: Values are the median (25th–75th percentiles).

Abbreviations: SAPS II, Simplified Acute Physiology Score II; APACHE II, Acute Physiology and Chronic Health Evaluation II; SOFA, sepsis‐related organ failure assessment; TISS, therapeutic intervention scoring system; CRP, C‐reactive protein; POD1, postoperative day 1; ICU, intensive care unit; LOS, length of stay.

The patient demographics and outcomes of 90‐day survivors and non‐survivors are presented in Table 3. The 90‐day survivors were younger and they had higher SAPS II, Apache II, SOFA, and TISS scores. Platelet count was lower within 90‐day non‐survivors. Results of the 90‐day non‐survivors were in line with those who died during the follow‐ up, but there was no significant difference between the indirect ICU admissions (Table 3).

**TABLE 3:** Comparison of 525 patients admitted to the ICU within 48 h of the EL

Characteristic	90‐day survivors *N* = 398	90‐day non‐survivors *N* = 127	*p*‐value
Age, years	64 (52–75)	73 (62–80)	<.001
Gender, male	223 (56.0)	75 (59.1)	.549
Operation duration (min)	196 (153–255)	208 (162–253)	.212
ASA	4 (3–4)	4 (3–4)	<.001
ASA 1	4 (1.0)	0	.257
ASA 2	26 (6.5)	2 (1.6)	.030
ASA 3	126 (31.7)	31 (24.4)	.120
ASA 4	186 (46.7)	63 (49.6)	.572
ASA 5	37 (9.3)	27 (21.3)	<.001
Outcomes			
SAPS II	34 (28–41)	46(35–58)	<.001
Apache II score	15 (11–19)	21 (16–25)	<.001
SOFA score on admission	5 (3–7)	7 (4–10)	<.001
SOFA, maximum score	6 (5–8)	10 (7–13)	<.001
Cumulative TISS score	118 (70–229)	141 (93–400)	.003
Median TISS score/day	6 (4–12)	18 (8–41)	<.001
CRP, POD1	191 (120–269)	185 (95–106)	.437
Platelet count, POD1	207 (141–288)	176 (77–248)	.001
Direct admission to ICU	241 (60.6)	82 (64.6)	.418
Indirect admission to ICU	94 (23.6)	33 (26.0)	.588
Admission from ward to ICU within 48 h of EL	63 (15.8)	12 (9.4)	.074
ICU LOS (days)	2.5 (1.3–4.9)	2.6 (1.3–8.7)	.323
Hospital LOS (days)	20 (11–37)	12 (5–23)	<.001
Invasive ventilation in ICU	309 (77.6)	112 (88.2)	.009
Duration of invasive ventilation (h)	14.7 (4.5–39.0)	32.6 (12.0–103.7)	<.001

*Note*: Values are the number (%) or the median (25th–75th percentiles), as indicated.

Abbreviations: ASA, American Society of Anesthesiologists; SAPS II, Simplified Acute Physiology Score II; APACHE II, Acute Physiology and Chronic Health Evaluation II; SOFA, sepsis‐related organ failure assessment; TISS, therapeutic intervention scoring system; CRP, C‐reactive protein; POD1, postoperative day 1; ICU, intensive care unit; LOS, length of stay.

According to the Cox regression model, admission from the PACU to the ICU, age, ASA, APACHE‐score, and CRP and platelet count of the first postoperative day were associated with death during the follow‐up (Table 4). The mortality in different time‐points is presented in Table 5.

**TABLE 4:** Variables associated with death during the follow‐up in the study population analyzed with the Cox regression model

	OR (95% Cl)	*p*‐value
Direct admission to ICU	1	
Admission from PACU to ICU	1.427 (1.059–1.923)	.020
Admission from ward to ICU	1.022 (0.691–1.513)	.912
Age	1.035 (1.025–1.045)	<.001
CRP, POD1	0.998 (0.997–1.000)	.008
Platelet count, POD1	0.999 (0.998–1.000)	.047
APACHE II score	1.065 (1.042–1.088)	<.001

Abbreviations: ICU, intensive care unit; PACU, post‐anesthesia care unit; CRP, C‐reactive protein; POD, postoperative day 1; APACHE II, Acute Physiology and Chronic Health Evaluation II.

**TABLE 5:** The mortality rates for 525 patients admitted to the ICU within 48 h of the EL

	All patients (*n* = 525)
Hospital mortality	70 (13.3)
30‐D mortality	91 (17.3)
90‐D mortality	127 (24.2)
One‐year mortality	173 (33.0)
Five‐year mortality	306 (58.3)

**FIGURE 1:**
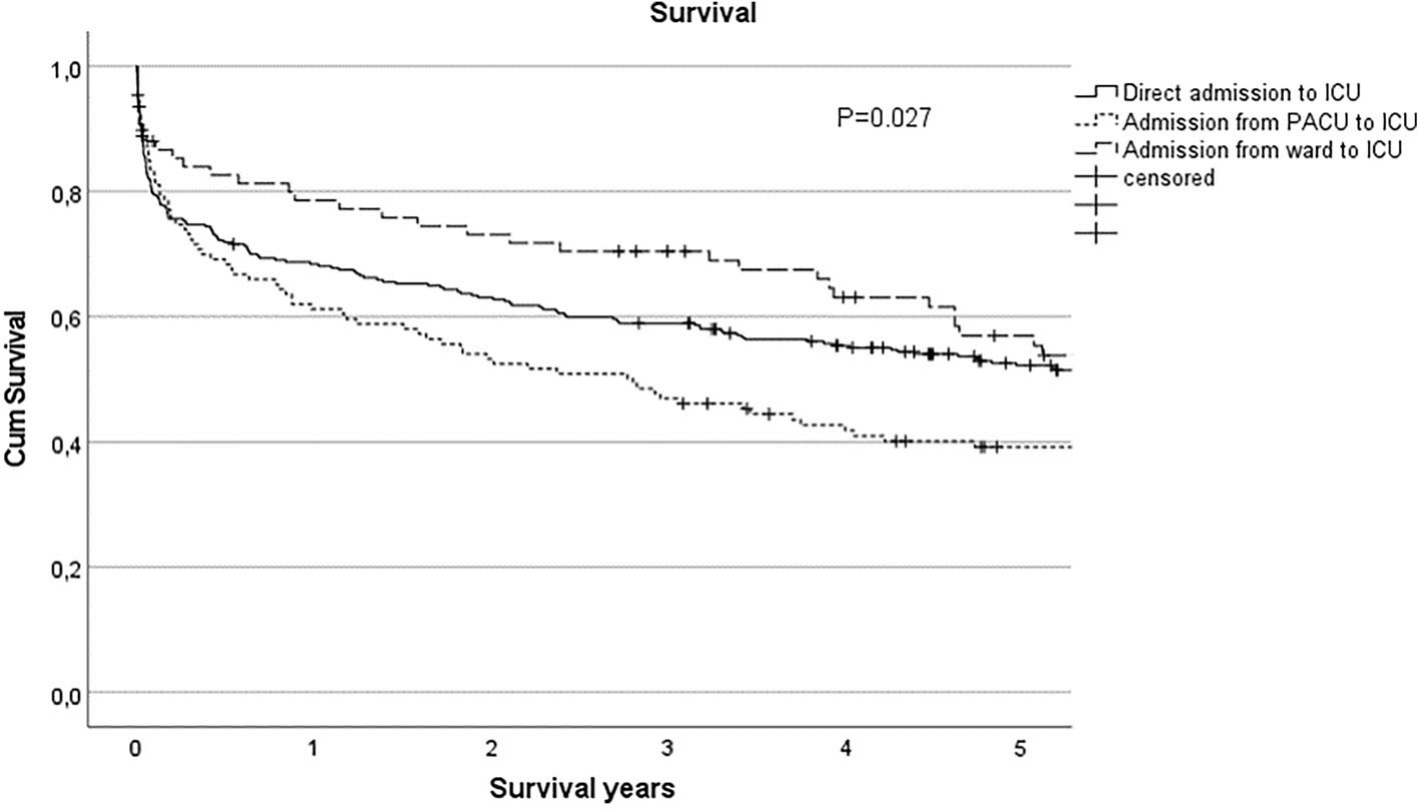
Kaplan–Meier survival curves for different ICU admission groups

Survival of the study population is presented in Figure 1. Patients with indirect ICU admission had highest long‐term mortality. Admission from PACU to ICU after EL associates with 40% 5‐year survival.

## 4 DISCUSSION

The main findings of the present study were that advancing age, lower CRP levels and platelet count on POD1, increasing APACHE II‐score, and indirect ICU admission were associated with death during the 5‐year follow‐up after EL. Additionally, we found that the 5‐year mortality after EL was high in patients admitted to the ICU within 48 h from surgery; over 50% of the population had died after 5 years of follow‐up.

Our results showed that the non‐survivors were more seriously ill compared to the survivors as demonstrated by the higher SOFA and APACHE scores. Most patients were assessed to be in a critical condition and required an ICU admission immediately after the EL, while the rest of the patients were admitted to the PACU to be later discharged to the surgical ward. A part of the patients primarily admitted to the PACU stayed there nearly 12 h but failed to achieve a clinical condition suitable for discharge to the ward and therefore were admitted to an ICU. Most of the patients coming from the PACU to the ICU received invasive ventilation during the ICU admission, indicating that a respiratory failure was the most dominant organ dysfunction leading to the ICU admission. The ICU admissions that were premeditated directly after EL may have had entailed a more straightforward weaning process from mechanical ventilation that might have shortened the duration of the respiratory support. The PACU care can include an ICU‐level mechanical ventilation and a hemodynamic support, but otherwise the PACU care is not as comprehensive as the care in the ICU. Also, the ICUs are generally better resourced in terms of nurse‐to‐patient ratios. However, these factors are unlikely to explain the difference in the long‐term mortality. The difference is easily explained by the age and comorbidities; one could hypothesize that the limited physical resources of these patients prevented them to recover during the immediate postoperative phase and this lack of capacity turns to a higher mortality during the follow‐up. Interestingly, an ICU admission from the surgical ward to the ICU was not associated with a poor outcome. These patients had achieved a clinical condition good enough to manage in the ward at first place but deteriorated later as a consequent on possible postoperative complications. It has been shown in other patient groups that especially medical postoperative complications are associated with poor outcomes.^13^, ^14^

Interestingly, in the first postoperative day, CRP values were lower in the non‐survivors compared to the survivors. This may reflect that the non‐survivors were more ill, which is supported by the lower platelet count. Preoperative sepsis is a risk factor for death after an EL.^15^ The CRP is an acute‐phase protein produced by the liver in response to various cytokines, including interleukin (IL)‐6, IL‐1, and tumor necrosis factor (TNF)‐alpha during acute injury, infections, inflammatory stimuli, and malignant disease.^16^ We measured only indirect markers of proinflammation so we are unable to assess the anti‐ inflammatory response to the critical illness. One explanation could be a more impaired immunological response due to frailty and co‐morbidities, such as malignancies, in non‐survivors. However, this finding needs further examination and we are not able to confirm or exclude this hypothesis in this study.

This study explored better short‐term outcomes (30‐ and 90‐day mortality [17.3%–24.2%]) than some previous studies reporting the short‐term mortality of 25.6–48.2%.^1^, ^3^, ^4^, ^17^ One study has reported lower 30‐ and 90‐day mortality rates after EL with a direct ICU admission (15.9% and 20.5%).^18^ This study was the first to report the 5‐year survival rates of patients admitted to the ICU within 48 h after an EL. Although the 30‐ and 90‐day mortality rates were lower in the present study compared to the previous studies, our 1‐year and 5‐year mortality rates were very high and in line with the other studies. According to the Finnish Cancer Registry,^19^ the reported 5‐year survival of the patients with colorectal carcinoma (64.2%) was better than the rates we found for the patients admitted to the ICU within 48 h after an EL (41.7%). The overall mortality rates that we found after an EL are unthinkable for any common major elective surgery.

In general, patients undergoing EL tend to be old, with many comorbidities, and they are at a high risk of postoperative complications.^1^, ^2^, ^4^ The patients over 65 years of age represent the most rapidly growing age group.^20^ In the present study, the non‐survivors were older and had more often malignancies than the survivors. Neither an ICU admission nor surgery are decisive factors in determining whether a single patient lives for five additional years after an EL. The National Emergency Laparotomy Audit (NELA) reported that the patients over 70 years old were 1.6‐fold more likely to be admitted directly to the ICU than the patients under 50 years old.^21^ Previous reports have shown that a delay in a post‐surgery ICU admission for the patients that were critically ill was associated with an increased mortality.^22^ Emergency abdominal surgery procedures account for more than 80% of the national burden associated with all emergency general surgery‐related inpatient costs.^23^ In the United States, the emergency general surgery accounted for 2.6 million hospitalizations, which cost $28.4 billion in 2010.^20^ Those costs are projected to increase by 45%, to $41.2 billion, by 2060.^20^ The early recognition and management of postoperative complications can reduce the mortality and the costs of post‐EL operations.^24^

The possible risk assessment tools available for the patients undergoing an EL include APACHE II and the ASA classification, which are easily accessible and widely used. APACHE II is an excellent tool for assessing individual risk in patients undergoing an EL, a fact that emerged also in this study.^9^ The relationship between a higher ASA classification score and a poor outcome has been shown previously, despite the fact that ASA is a highly subjective estimation of the patient condition.^21^, ^25^ Davenport et al reported that ASA is strong predictor of outcomes, but still the NSQIP surgical risk calculator without ASA is better predictor than ASA alone.^26^ The NELA risk adjustment model has demonstrated an excellent performance in predicting short‐term postoperative mortality after EL.^27^ The patients that require an ICU admission should be identified early, because an early ICU admission is more likely to produce positive outcomes.^28^, ^29^ The emergency laparotomy pathway quality improvement care (ELPQuiC) bundle includes also early ICU admission and it has been shown that ELPQuiC bundle reduced risk‐adjusted mortality after EL.^30^ Standardization of care and using simple evidence‐based guidelines improve EL patients’ prognosis.^30^

This study had several limitations. The main limitation was the retrospective study design. Additionally, the single‐center study design might restrict the generalization of our results. Moreover, due to the retrospective study design, we could not include data about how the discharge strategy was determined or which factors might have influenced to the strategy, such as the rates of ICU or PACU bed occupation. Moreover, we were not able to include data for the causes of the admissions from a ward to the ICU, and therefore we can only hypothesize the role of the postoperative complications in this patient group. The role of the non‐surgery‐related and the non‐acute abdomen‐related causes of admissions among those admitted from the PACU to the ICU cannot be covered in this study setting. Part of the high mortality could be explained by the developing medical complications, which have shown to be associated with poor long‐term outcome in other patient groups.^13^, ^14^

## 5 CONCLUSION

Age, higher APACHE II sore, lower CRP and platelet count of POD1, and the admission from the PACU to the ICU were associated with worse prognosis after an EL. The 1‐ and 5‐year mortality rates after EL were high.

## FUNDING INFORMATION

This research did not receive any specific grant from agencies in the public, commercial, or not‐for‐profit sectors.

## CONFLICT OF INTEREST

Aura T Ylimartimo, Marjo Koskela, Sanna Lahtinen, Timo Kaakinen, Merja Vakkala, and Janne Liisanantti declare that they have no conflict of interest.

## References

[1] Vester‐Andersen M , Lundstrøm LH , Møller MH , Waldau T , Rosenberg J , Møller AM . Mortality and postoperative care pathways after emergency gastrointestinal surgery in 2904 patients: a population‐based cohort study. Br J Anaesth. 2014;112:860‐870. 24520008 10.1093/bja/aet487

[2] Sørensen LT , Malaki A , Wille‐Jørgensen P , et al. Risk factors for mortality and postoperative complications after gastrointestinal surgery. J Gastrointest Surg. 2007;11:903‐910. 17468915 10.1007/s11605-007-0165-4

[3] Saunders DI , Murray D , Pichel AC , Varley S , Peden CJ . Variations in mortality after emergency laparotomy: the first report of the UK Emergency Laparotomy Network. Br J Anaesth. 2012;109:368‐375. 22728205 10.1093/bja/aes165

[4] Awad S , Herrod PJJ , Palmer R , et al. One‐ and two‐year outcomes and predictors of mortality following emergency laparotomy: a consecutive series from a United Kingdom teaching hospital. World J Surg. 2012;36:2060‐2067. 22538391 10.1007/s00268-012-1614-0

[5] Peponis T , Bohnen JD , Sangji NF , et al. Does the emergency surgery score accurately predict outcomes in emergent laparotomies? Surgery. 2017;162:445‐452. 28554491 10.1016/j.surg.2017.03.016

[6] Havens, J.M. , Peetz, A.B. , Do, W.S. , et al. The excess morbidity and mortality of emergency general surgery. J Trauma Acute Care Surg, 2015; 78(2), 306–311. ‐ PubMed ‐ NCBI. https://www-ncbi-nlm-nih-gov.pc124152.oulu.fi:9443/pubmed/25757115 25757115 10.1097/TA.0000000000000517

[7] Sharoky, C.E. , Bailey, E.A. , Sellers, M.M. , Kaufman, E.J. , Sinnamon, A.J. , Wirtalla, C.J. , Holena, D.N. and Kelz, R.R. Outcomes of hospitalized patients undergoing emergency general surgery remote from admission. Surgery, 2017; 162(3), 612–619. ‐PubMed ‐ NCBI. https://www-ncbi-nlm-nih-gov.pc124152.oulu.fi:9443/pubmed/28689604 28689604 10.1016/j.surg.2017.05.008

[8] Jeppesen MM , Thygesen LC , Ekeloef S , Gögenur I . A nationwide cohort study of short‐ and long‐term outcomes following emergency laparotomy. Dan Med J. 2019;66(1):1‐6. 30573005

[9] Oliver CM , Walker E , Giannaris S , Grocott MPW , Moonesinghe SR . Risk assessment tools validated for patients undergoing emergency laparotomy: a systematic review. Br J Anaesth. 2015;115:849‐860. 26537629 10.1093/bja/aev350

[10] Pędziwiatr M , Mavrikis J , Witowski J , et al. Current status of enhanced recovery after surgery (ERAS) protocol in gastrointestinal surgery. Med Onco (Northwood, London, England). 2018;35:95. 10.1007/s12032-018-1153-0 PMC5943369 29744679

[11] Tengberg LT , Bay‐Nielsen M , Bisgaard T , Cihoric M , Lauritsen ML , Foss NB . Multidisciplinary perioperative protocol in patients undergoing acute high‐risk abdominal surgery. Br J Surg. 2017;104:463‐471. 28112798 10.1002/bjs.10427

[12] Chana P , Joy M , Casey N , et al. Cohort analysis of outcomes in 69 490 emergency general surgical admissions across an international benchmarking collaborative. BMJ Open. 2017;7:e014484. 10.1136/bmjopen-2016-014484 PMC5353261 28274969

[13] Junttola U , Lahtinen S , Liisanantti J , Vakkala M , Kaakinen T , Isokangas J‐M . Medical complications and outcome after endovascular therapy for acute ischemic stroke. Acta Neurol Scand. 2021;144:623‐631. 34263446 10.1111/ane.13501

[14] Lahtinen S , Koivunen P , Ala‐Kokko T , et al. Complications and outcome after free flap surgery for cancer of the head and neck. Br J Oral Maxillofac Surg. 2018;56:684‐691. 30107953 10.1016/j.bjoms.2018.07.009

[15] Haskins IN , Maluso PJ , Schroeder ME , et al. A calculator for mortality following emergency general surgery based on the American College of Surgeons National Surgical Quality Improvement Program database. J Trauma Acute Care Surg. 2017;82:1094‐1099. 28328681 10.1097/TA.0000000000001451

[16] Donlon NE , Mohan H , Free R , et al. Predictive value of CRP/albumin ratio in major abdominal surgery. Ir J Med Sci. 2020;189:1465‐1470. 32361882 10.1007/s11845-020-02238-y

[17] Watt DG , Wilson MSJ , Shapter OC , Patil P . 30‐day and 1‐year mortality in emergency general surgery laparotomies: an area of concern and need for improvement? Eur J Trauma Emerg Surg. 2015;41:369‐374. 26037986 10.1007/s00068-014-0450-3

[18] Oliver CM , Bassett MG , Poulton TE , et al. Organisational factors and mortality after an emergency laparotomy: multilevel analysis of 39 903 National Emergency Laparotomy Audit patients. Br J Anaesth. 2018;121:1346‐1356. 30442263 10.1016/j.bja.2018.07.040

[19] Finish Cancer Registy . Finish Cancer Registy. 2019. https://syoparekisteri.fi/tilastot/tautitilastot

[20] Ogola GO , Gale SC , Haider A , Shafi S . The financial burden of emergency general surgery: national estimates 2010 to 2060. The Journal of Trauma and Acute Care Surgery. 2015;79:444‐448. 26307879 10.1097/TA.0000000000000787

[21] LRN Project Team . The fifth Patient Report of the NELA 2019‐ Full Patient Report.pdf. 2019. http://www.nela.org.uk/reports

[22] Bing‐Hua YU . Delayed admission to intensive care unit for critically surgical patients is associated with increased mortality. Am J Surg. 2014;208:268‐274. 24480235 10.1016/j.amjsurg.2013.08.044

[23] Scott JW , Olufajo OA , Brat GA , et al. Use of National Burden to define operative emergency general surgery. JAMA Surg. 2016;151:e160480. 27120712 10.1001/jamasurg.2016.0480

[24] Shah AA , Haider AH , Zogg CK , et al. National estimates of predictors of outcomes for emergency general surgery. J Trauma Acute Care Surg. 2015;78:482‐491. 25710417 10.1097/TA.0000000000000555

[25] Lees MC , Merani S , Tauh K , Khadaroo RG . Perioperative factors predicting poor outcome in elderly patients following emergency general surgery: a multivariate regression analysis. Can J Surg. 2015;58:312‐317. 26204143 10.1503/cjs.011614 PMC4599992

[26] Davenport DL , Bowe EA , Henderson WG , Khuri SF , Mentzer RM . National Surgical Quality Improvement Program (NSQIP) risk factors can be used to validate American Society of Anesthesiologists Physical Status Classification (ASA PS) levels. Ann Surg. 2006;243:636‐641. discussion 641‐4. 16632998 10.1097/01.sla.0000216508.95556.cc PMC1570549

[27] Eugene N , Oliver CM , Bassett MG , et al. Development and internal validation of a novel risk adjustment model for adult patients undergoing emergency laparotomy surgery: the National Emergency Laparotomy Audit risk model. Br J Anaesth. 2018;121:739‐748. 30236236 10.1016/j.bja.2018.06.026

[28] Cardoso LTQ , Grion CMC , Matsuo T , et al. Impact of delayed admission to intensive care units on mortality of critically ill patients: a cohort study. Crit Care. 2011;15:R28. 21244671 10.1186/cc9975 PMC3222064

[29] Flabouris A , Jeyadoss J , Field J , Soulsby T . Direct and delayed admission to an intensive care or high dependency unit following discharge from the emergency department: associated patient characteristics and hospital outcomes. Crit Care Resusc. 2012;14:191‐197. 22963213

[30] Huddart S , Peden CJ , Swart M , et al. Use of a pathway quality improvement care bundle to reduce mortality after emergency laparotomy. Br J Surg. 2015;102:57‐66. 25384994 10.1002/bjs.9658 PMC4312892

